# Data on lung and intestinal microbiome after air pollution exposure in ageing rats

**DOI:** 10.1016/j.dib.2023.109004

**Published:** 2023-02-23

**Authors:** Vincent Laiman, Yu-Chun Lo, Hsin-Chang Chen, Tzu-Hsuen Yuan, Ta-Chih Hsiao, Jen-Kun Chen, Ching-Wen Chang, Ting-Chun Lin, Ssu-Ju Li, You-Yin Chen, Didik Setyo Heriyanto, Kian Fan Chung, Kai-Jen Chuang, Kin-Fai Ho, Jer-Hwa Chang, Hsiao-Chi Chuang

**Affiliations:** a International Ph.D. Program in Medicine, College of Medicine, Taipei Medical University, Taipei, Taiwan; b Department of Anatomical Pathology, Faculty of Medicine, Public Health, and Nursing, Universitas Gadjah Mada – Dr. Sardjito Hospital, Yogyakarta, Indonesia; c Ph.D. Program in Medical Neuroscience, College of Medical Science and Technology, Taipei Medical University, Taipei, Taiwan; d Department of Chemistry, College of Science, Tunghai University, Taichung, Taiwan.; e Department of Health and Welfare, College of City Management, University of Taipei, Taipei, Taiwan; f Graduate Institute of Environmental Engineering, National Taiwan University, Taipei, Taiwan; g Institute of Biomedical Engineering & Nanomedicine, National Health Research Institutes, Miaoli, Taiwan; h Industrial Ph.D. Program of Biomedical Science and Engineering, National Yang Ming Chiao Tung University, Taipei, Taiwan; i Department of Biomedical Engineering, National Yang Ming Chiao Tung University, Taipei, Taiwan; j National Heart and Lung Institute, Imperial College London, London, UK; k School of Public Health, College of Public Health, Taipei Medical University, Taipei, Taiwan; l Department of Public Health, School of Medicine, College of Medicine, Taipei Medical University, Taipei, Taiwan; m School of Public Health and Primary Care, the Chinese University of Hong Kong, Hong Kong; n School of Respiratory Therapy, College of Medicine, Taipei Medical University, 250 Wuxing Street, Taipei 11031, Taiwan; o Division of Pulmonary Medicine, Departments of Internal Medicine, Wan Fang Hospital, Taipei Medical University, Taipei, Taiwan; p Division of Pulmonary Medicine, Department of Internal Medicine, Shuang Ho Hospital, Taipei Medical University, New Taipei City, Taiwan; q Cell Physiology and Molecular Image Research Center, Wan Fang Hospital, Taipei Medical University, Taipei, Taiwan; r Graduate Institute of Medical Sciences, College of Medicine, Taipei Medical University, Taipei, Taiwan

**Keywords:** Air pollution, Lung microbiome, Intestinal microbiome, PM_2.5_

## Abstract

Air pollution has been linked to respiratory diseases, and urban air pollution can be attributed to a number of emission sources. The emitted particles and gases are the primary components of air pollution that enter the lungs during respiration. Particulate matter with an aerodynamic diameter of ≤ 2.5 µm (PM_2.5_) can deposit deep into the respiratory tract via inhalation and has been proposed as a causative agent for adverse respiratory health. In addition, the lung contains a diverse microbial community (microbiome) that maintains normal homeostasis and is significantly altered in a variety of pulmonary disorders. Air pollution, specifically PM_2.5_, has previously been shown to significantly alter the composition of the lower airway microbiome, which has been linked to decreased lung function in chronic obstructive pulmonary disease (COPD) patients. Surprisingly, the intestinal microbiome has also been implicated in the modulation of pulmonary inflammatory diseases. Therefore, dysbiosis of the lung and intestinal microbiomes pose significant negative effects on human health.

This dataset describes the microbial community profiles of the lungs and intestines of ageing rats exposed to ambient unconcentrated traffic-related air pollution for three months. The whole-body exposure system was equipped with and without high efficiency particulate air (HEPA) filtration (gaseous vs. PM_2.5_ pollution). The data can provide valuable information on lung and intestinal microbiome changes, including that which was only found after traffic-related air pollution exposure.

**Specifications Table**

**Table:** 

Subject	Microbiology: microbiome

Specific subject area	Investigation of the changes in lung and intestinal microbiome in ageing rats after air pollution exposure
Type of data	Table
How the data were acquired	Illumina MiSeq platform
Data format	Raw and analyzed
Description of data collection	Lung and fecal samples were collected from ageing rats in control, high-efficiency particulate air (HEPA) filter, and particulate matter with aerodynamic diameter of ≤ 2.5 µm (PM_2.5_) exposure groups. Total DNA was extracted from the samples, and the 16S rDNA gene sequencing was performed using the Illumina MiSeq platform.
Data source location	City/Town/Region: Taipei Country: Taiwan Latitude and longitude: 25°1′5.2176′'N, 121°32′17.8548′'E
Data accessibility	Repository name: Mendeley Data Data identification number: 10.17632/26td6trhmg.3 Direct URL to data: http://dx.doi.org/10.17632/26td6trhmg.3
Related research article	V. Laiman, Y.-C. Lo, H.-C. Chen, T.-H. Yuan, T.-C. Hsiao, J.-K. Chen, C.-W. Chang, T.-C. Lin, S.-J. Li, Y.-Y. Chen, D.S. Heriyanto, K.F. Chung, K.-J. Chuang, K.-F. Ho, J.-H. Chang, H.-C. Chuang, Effects of antibiotics and metals on lung and intestinal microbiome dysbiosis after sub-chronic lower-level exposure of air pollution in ageing rats, Ecotoxicology and Environmental Safety 246 (2022). https://doi.org/10.1016/j.ecoenv.2022.114164

## Value of the Data

• The data provides information on lung and intestinal microbiome changes in phylum and family level from ageing rats, including that which was only found after traffic-related air pollution exposure [1].
• The data could be useful for the comparative analysis of the lung and intestinal microbiome profiles of rats with traffic-related air pollution exposure in Taipei, Taiwan compared to other region.
• The data could be useful for studies on the mechanism of lung and intestinal microbiome dysbiosis by air pollution exposure.
• This data will be useful for subsequent studies to investigate the influence of air pollution components, including the chemical and metal components, and gaseous pollutants on the composition of lung and intestinal microbiome.

## Objective

This study aimed to demonstrate that exposure to traffic-related air pollution for three months alters the lung and intestinal microbiome in ageing rats.

## 1 Data Description

**Fig. 1:**
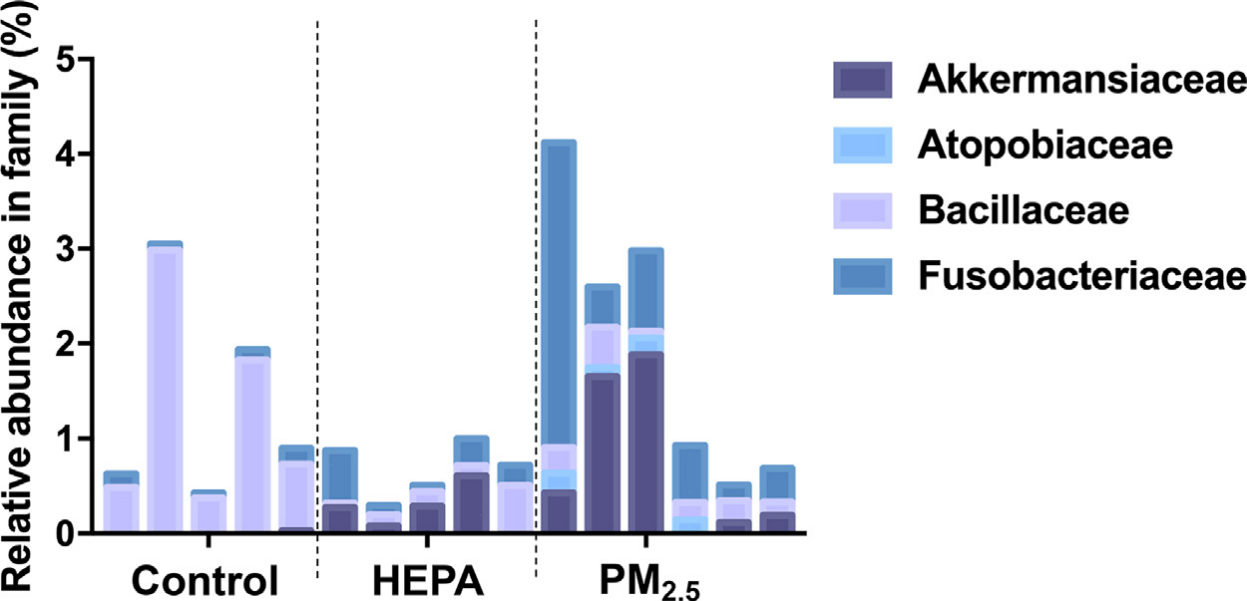
Relative abundance of lung microbiome analysis in family level between control, control, high efficiency-filtered air (HEPA) filter, and particulate matter with an aerodynamic diameter of ≤ 2.5 µm (PM_2.5_) groups.

**Fig. 2:**
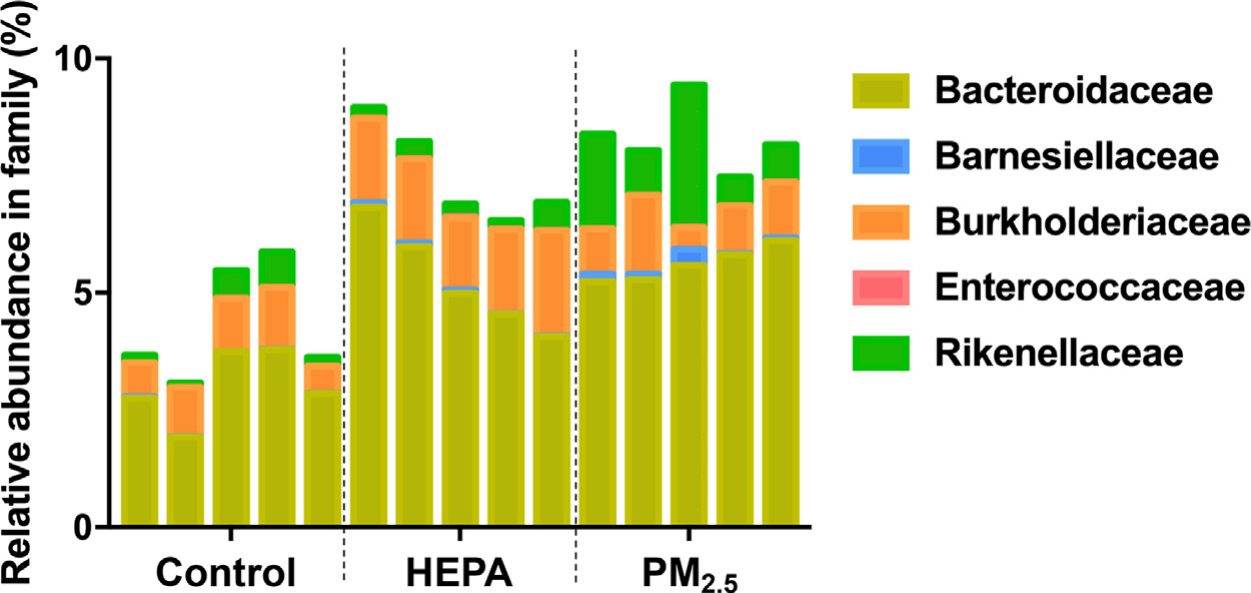
Relative abundance of intestinal microbiome analysis in family level between control, control, high efficiency-filtered air (HEPA) filter, and particulate matter with an aerodynamic diameter of ≤ 2.5 µm (PM_2.5_) groups.

The raw datasets contain 16S ribosomal (r)DNA gene sequences derived from lung and fecal samples collected from ageing rats exposed to traffic-related air pollution for three months [1,2]. The rats were divided into three groups: control, high efficiency-filtered air (HEPA) filter, and particulate matter with an aerodynamic diameter of ≤ 2.5 µm (PM_2.5_) groups. The alpha diversity indices (Chao 1) of each group was identified. In the lung microbiome, nine phyla of the samples were identified, included Actinobacteria, Bacteroidetes, Epsilonbacteraeota, Firmicutes, Fusobacteria, Proteobacteria, Synergistetes, Tenericutes, and Verrucomicrobia, with the three most abundant phylum were Proteobacteria, Firmicutes, and Bacteroidetes (Table 1). The Proteobacteria was found highest in relative abundance in control, followed by HEPA, and PM_2.5_ groups (89.05 %, 85.78 %, and 79.13 %, respectively). In contrast, Firmicutes and Bacteroidetes were found highest in PM_2.5_ (9.79 % and 8.01 %), followed by HEPA (7.05 % and 6.04 %) and control groups (5.70 % and 4.09 %). Family profiles of the lung microbiome between the groups revealed the presence of four families: Akkermansiaceae, Atopobiaceae, Bacillaceae, and Fusobacteriaceae (Fig. 1). Bacteria belonging to the Bacillaceae family (1.27%) were the most abundant in control group. In contrast, Akkermansiaceae was the most abundant in HEPA group (0.25%) and Fusobacteriaceae were the most abundant in PM_2.5_ group (0.93%). Of note, the Atopobiaceae was only found in lungs of rats in PM_2.5_ group. In the intestinal microbiome, Actinobacteria, Bacteroidetes, Deferribacteres, Epsilonbacteraeota, Firmicutes, Fusobacteria, Patescibacteria, Proteobacteria, Tenericutes, and Verrucomicrobia were identified in the phylum level, with the two most abundant phylum were Firmicutes and Bacteroidetes (Table 2). Firmicutes was found highest in relative abundance in control, followed by HEPA, and PM_2.5_ groups (43.51 %, 38.79 %, and 37.42 %, respectively). Bacteroidetes, on the other hand, were found highest in PM_2.5_, followed by HEPA and control groups (55.45 %, 51.25 %, and 49.73%, respectively). A comparison of family profiles of the intestinal microbiome between the groups showed that there were five family, Bacteroidaceae, Barnesiellaceae, Burkholderiaceae, Enterococcaceae, and Rikenellaceae present (Fig. 2). Among them, bacteria belonging to Bacteroidaceae family were the most abundant, with the highest found in PM_2.5_, followed by HEPA and control groups (5.61 %, 5.28 %, and 3.01 %, respectively).

**Table 1:** Relative abundance of the lung microbiome in phylum level between control, high efficiency-filtered air (HEPA) filter, and particulate matter with an aerodynamic diameter of ≤ 2.5 µm (PM_2.5_) groups.

Phylum	Control	HEPA	PM_2.5_
Actinobacteria	0.95 %	0.49 %	1.19 %
Bacteroidetes	4.09 %	6.04 %	8.01 %
Epsilonbacteraeota	0.03 %	0.06 %	0.05 %
Firmicutes	5.70 %	7.05 %	9.79 %
Fusobacteria	0.16 %	0.24 %	0.99 %
Proteobacteria	89.05 %	85.78 %	79.13 %
Synergistetes	0.00 %	0.00 %	0.04 %
Tenericutes	0.01 %	0.02 %	0.05 %
Verrucomicrobia	0.01 %	0.25 %	0.71 %

**Table 2:** Relative abundance of the intestinal microbiome in phylum level between control, high efficiency-filtered air (HEPA) filter, and particulate matter with an aerodynamic diameter of ≤ 2.5 µm (PM_2.5_) groups.

Phylum	Control	HEPA	PM_2.5_
Actinobacteria	0.84 %	1.15 %	0.63 %
Bacteroidetes	49.73 %	51.25 %	55.45 %
Deferribacteres	0.01 %	0.00 %	0.03 %
Epsilonbacteraeota	0.05 %	0.04 %	0.03 %
Firmicutes	43.51 %	38.79 %	37.42 %
Fusobacteria	0.45 %	0.71 %	0.41 %
Patescibacteria	2.42 %	2.34 %	2.86 %
Proteobacteria	2.00 %	4.98 %	2.15 %
Tenericutes	0.01 %	0.00 %	0.01 %
Verrucomicrobia	0.98 %	0.74 %	1.01 %

## 2 Experimental Design, Materials and Methods

Male 1.5-year-old Fischer 344 ageing rats (National Laboratory Animal Center, Taipei, Taiwan) were housed at constant temperature of 22 ± 2 °C and relative humidity (RH) of 55% ± 10% with 12:12-h light: dark cycle. Ageing rats housed in the animal center and supplied with HEPA-filtered clean air served as control group. In the air pollution exposure groups, ageing rats were continually exposed to ambient unconcentrated traffic-related air pollution for 3 months (24 h/day) using whole-body exposure system equipped with and without HEPA filtration (gaseous vs. gaseous and PM_2.5_ pollution). The system for the whole-body exposure has been described previously [3,4]. Briefly, an omnidirectional particulate matter inlet was installed on the roof of the animal housing with continuous ambient air passing into the chamber. The airflow was then introduced into each cage of the whole-body exposure system. The air used in the whole-body exposure system was sourced from a nearby highway and expressway in a traffic-heavy urban area (Taipei, Taiwan; 25°1′5.2176′'N, 121°32′17.8548′'E). The concentrations and characteristics of air pollution for the study period have been previously reported [2]. In summary, continuous monitoring of ambient air pollution characteristics in the exposure chamber revealed an average of 8.7 ± 4.2 µg/m^3^ ambient PM_2.5_ mass concentration, with geometric mean diameter of 64.5 ± 6.8 nm, and particle number concentration of 6460.5 ± 2086.2 particles/cm^3^. The gaseous pollutant profile was obtained from the Guting air quality monitoring station of the Taiwan Environmental Protection Administration (EPA). The gaseous pollution data during the study period revealed carbon monoxide (CO) of 0.3 ± 0.1 ppm, sulfur dioxide (SO_2_) of 1.6 ± 0.4 ppb, nitrogen dioxide (NO_2_) of 10.6 ± 4.2 ppb, and ozone (O_3_) levels of 24.7 ± 10.5 ppb. The temperature was 29.1 ± 2.4 °C with relative humidity of 73.4 % ± 4.7 %. The lung tissues and fecal samples were collected after 3 months of exposure and were stored in -80°C until further processing.

About 10 mg of lung samples and 220 mg of fecal samples underwent DNA extraction using QIAamp DNeasy Blood & Tissue Kits and QIAamp DNA Stool Mini Kit (Qiagen, Hilden, Germany). The minimum final concentration of intestinal and lung bacterial DNA samples was 5 ng/µL, and all DNA samples were stored at -80°C. Universal 16S ribosomal (r)RNA gene primers V3 (341F, 5’-CCTACGGGNGGCWGCAG-3’) and V4 (805R, 5’-GACTACHVGGGTATCTAATCC-3’) were recommended and designed by Illumina (https://support.illumina.com/downloads/16s_metagenomic_sequencing_library_preparation.html). These two primers involved overhang adapter sequences in the forward (5’-TCGTCGGCAGCGTCAGATGTGTATAAGAGACAG-3′) and reverse (5’-GTCTCGTGGGCTCGGAGATGTGTATAAGAGACAG-3′) primers and amplified the targeted sequence of the bacterial 16S rDNA gene [5]. In addition, a limited cycle polymerase chain reaction (PCR) amplified the V3-V4 region of the bacterial 16S rDNA gene to construct the amplicon library. The sequencing libraries, Illumina sequencing adapters, and dual-index barcodes were attached to the amplicon library. To ensure that the amount was sufficient to attach 16S rDNA, the quantity and quality of the sequencing libraries were confirmed by a QSep100 analyzer (BiOptic, New Taipei City, Taiwan). The v3 chemistry generated paired-end reads of 300 bases in length to normalize the libraries, pool the library in an equimolar ratio, and sequence them on Illumina MiSeq.

After 16S rDNA sequencing, the universal primer sequence and low-quality reads were removed. The following process and analysis were executed with the phyloseq workflow of the DADA2 package (vers. 1.6) in *R* environment [6]. Functions of the DADA2 package included filtering, trimming, de-replication, and de-noising of the forward and reverse reads. After merging the processed overlapping paired-end reads, chimers were removed from the cleaned full-length amplicons. Taxonomic assignment of the inferred amplicon sequence variants (ASVs) was performed using the SILVA reference database (vers. 132) with minimum bootstrap confidence of 80 [7]. Multiple sequences were aligned to ASVs with the DECIPHER package (vers. 2.6.0), and RAxML (vers. 8.2.11) was used to construct a phylogenetic tree. The phyloseq package (vers. 1.22.3) created a phyloseq object for downstream bacterial community analyses based on the frequency table, taxonomy, and phylogenetic tree information. Figures were created using GraphPad v. 9 (San Diego, CA, USA) for macOS.

## Ethics Statements

This study was conducted in compliance with the Animal and Ethics Review Committee of the Laboratory Animal Center at Taipei Medical University (Taipei, Taiwan; IACUC: LAC-2019-0424).

## CRediT authorship contribution statement

**Vincent Laiman:** Data curation, Writing – original draft. **Yu-Chun Lo:** Conceptualization, Methodology, Software. **Hsin-Chang Chen:** Conceptualization, Methodology, Software. **Tzu-Hsuen Yuan:** Data curation, Writing – original draft. **Ta-Chih Hsiao:** Data curation, Writing – original draft. **Jen-Kun Chen:** Data curation, Writing – original draft. **Ching-Wen Chang:** Data curation, Writing – original draft. **Ting-Chun Lin:** Data curation, Writing – original draft. **Ssu-Ju Li:** Data curation, Writing – original draft. **You-Yin Chen:** Visualization, Investigation. **Didik Setyo Heriyanto:** Writing – review & editing. **Kian Fan Chung:** Writing – review & editing. **Kai-Jen Chuang:** Software, Validation. **Kin-Fai Ho:** Software, Validation. **Jer-Hwa Chang:** Conceptualization, Methodology, Software, Supervision. **Hsiao-Chi Chuang:** Conceptualization, Methodology, Software, Supervision, Writing – review & editing.

## Declaration of Competing Interest

The authors declare that they have no known competing financial interests or personal relationships that could have appeared to influence the work reported in this paper.

## Data Availability

Data on Lung and Intestinal Microbiome in Ageing Rats exposed to Air Pollution (Original data) (Mendeley Data).
